# Erythropoietin promoted the proliferation of hepatocellular carcinoma through hypoxia induced translocation of its specific receptor

**DOI:** 10.1186/s12935-017-0494-7

**Published:** 2017-12-11

**Authors:** Shuo Miao, Su-Mei Wang, Xue Cheng, Yao-Feng Li, Qing-Song Zhang, Gang Li, Song-Qing He, Xiao-Ping Chen, Ping Wu

**Affiliations:** 1 Department of Pathophysiology, Tongji Medical College, Huazhong University of Science and Technology, Wuhan, 430030 China; 2 Department of Surgery, Tongji Hospital, Tongji Medical College, Huazhong University of Science and Technolgy, Wuhan, 430030 China; 3 Department of Surgery, Liyuan Hospital, Tongji Medical College, Huazhong University of Science and Technology, Wuhan, 430074 China; 4 Department of Hepatobiliary Surgery, The First Affiliated Hospital of Guangxi Medical University, Nanning, 530021 China; 5 Hepatic Surgery Center, Tongji Hospital, Tongji Medical College, Huazhong University of Science and Technolgy, Wuhan, 430030 China

**Keywords:** Erythropoietin, Erythropoietin receptor, Hepatocellular carcinoma, Hypoxia, Proliferation

## Abstract

**Background:**

Erythropoietin (EPO) is a hypoxia-inducible stimulator of erythropoiesis. Besides its traditional application in anemia therapy, it offers an effective treatment in the cancer patients, especially those who receive chemotherapy. Several reports indicated that it could promote the tumor cell proliferation through its specific receptor (EPOR). Unfortunately, the role of EPO/EPOR in hepatocellular carcinoma (HCC) progressing is still uncertain.

**Methods:**

Protein in tumor tissue from HCC patients or H22 tumor-bearing mice was detected with immunohistochemistry. Cells were cultured under 1% oxygen to establish hypoxia. RT-PCR and western blotting were used to measure mRNA and protein of EPO/EPOR, respectively. MTT, flow cytometry and PCNA staining were used to detect cell proliferation. Immunofluorescence staining was applied to study the expression and location of cellular EPOR. The EPOR binding studies were performed with ^125^I-EPO radiolabeling assay.

**Results:**

EPO and EPOR protein were up-regulated in HCC tissue of patients and H22-bearing mice. These were positively correlated with hypoxia-inducible factor -1 α and ki-67. Hypoxia up-regulated the expression of EPO and EPOR in HepG2 cells. It also induced the proliferation and increased the percentage of divided cells after 24, 48 and 72 h treatment. These were inhibited in cells pre-treated with 0.5 μg/mL soluble-EPOR. Immunofluorescence staining presented that EPOR was obviously translocated from nucleus to cytoplasm and membrane under hypoxia. EPOR binding activity was also increased after exposure to hypoxia. Recombinant human erythropoietin obviously elevated cell proliferation rate and the percentage of divided under hypoxia but not normoxia, which were also inhibited by soluble-EPOR.

**Conclusions:**

Our result indicated for the first time that EPO promoted the proliferation of HCC cells through hypoxia induced translocation of it specific receptor.

*Trial registration* TJC20141113, retrospectively registered

## Background

Hepatocellular carcinoma (HCC) is one of the most common cancers and the third most frequent cause of cancer-related death worldwide [1]. This situation is particularly concerning in China. Statistics have shown that the annual incidence of HCC in China alone contributes to 55% of global HCC cases. Previously, we have conducted a series of clinical and basic investigations into the mechanism and treatment of HCC [2–5]. Unfortunately, the tumor in most HCC patients is not suitable for surgical resection and carries a very poor prognosis. To these patients, conventional therapy provides very limited benefit and more effective systemic therapies are urgently needed.

Erythropoietin (EPO) is a single chain glycoprotein, with an approximate molecular weight of 30–34.0 kDa, which is produced by the kidneys and, to a lesser extent, the liver [6]. It can regulate the survival, proliferation and differentiation of erythrocytic progenitors and the maturation of erythrocytic precursors [7]. As the first identified hematopoietic factor, one of the key indication for its clinical use is in the management of severe anemia [8]. In addition, EPO had also been known for its angiogenic, anti-inflammatory, endothelial cell stabilization and neuroprotective effects [9–12].

Recently, EPO has drawn much attention in the field of cancer treatment, because anemia is a common complication in cancer patients, especially those who receive chemotherapy [13, 14]. Erythropoiesis-stimulating agents (ESA), such as recombinant human erythropoietin (rHuEPO), offer an effective treatment by decreasing the use of blood transfusion and improving disease-specific measures of life quality [15–18]. However, it cannot be ignored that there are still some potential risks of rHuEPO therapy in cancer patients. Beside its most common adverse effect to increasing venous thromboembolism [19], there is much more to be concerned regarding its direct influence on tumor progression such as in cervical carcinogenesis [20] and breast cancer [21]. Several clinical-trials even indicated impaired disease control or decreased survival in cancer patients treated with EPO, such as in head and neck cancer patients [22]. Based on current situation, it is quite necessary to conduct further exploration on the effect of EPO, both endogenous and exogenous, on tumor progress.

In order to make a better understanding of the function of EPO and its specific receptor (EPOR) in HCC, this study was performed to investigate their expression in the clinical sample, H22 tumor-bearing mice tissue and in vitro cultured HCC cells, and whether they were correlated with hypoxia and proliferation. Furthermore, the effect of hypoxia on the proliferation of human HCC cell line was studied. It is out of expectation that rHuEPO could promote cell proliferation under hypoxia but not under normoxia. For the first time, we presented the evidence that this effect of rHuEPO is dependent on hypoxia-induced membrane translocation of EPOR.

## Methods

### Patients and tumor samples

Twenty-eight pairs of tumors and adjacent non-tumor liver tissues (ANLTs) were collected from patients with HCC who had undergone surgical resection at the Hepatic Surgery Center, Tongji Hospital of Huazhong University of Science and Technology, Wuhan, China, between Jan 2013 and Dec 2015. All of the recruited patients in this study were not subjected to preoperative radiotherapy and/or chemotherapy. The HCC diagnosis was based on the histochemistry assay according to WHO criteria [23, 24]. Informed content was obtained, and access to human samples was carried out in accordance with the approved consent of the Ethics Committee of Tongji Hospital (Approval No. TJ-C20141113).

### IHC staining

Tissue sections (4-µm) from the clinical samples were deparaffinized, rehydrated and then heated in 10 mmol/L citrate buffer (pH 6.0) for antigen retrieval. Subsequently, the sections were washed in PBS, blocked with 10% normal goat serum for 30 min and incubated with primary antibodies to hypoxia inducible factor-1α (HIF-1α) (1:1000), EPO (1:500), EPOR (1:500), Ki67 (1:500) in a humidified chamber overnight at 4 °C. Immunodetection was performed using the Envision™ABC kit (GeneTech Co., Ltd., Shanghai, China). All samples were observed with a Leica DM4000B/M microscope (Leica Microsystems, Inc., Buffalo Grove, IL, USA). All antibodies were purchased from Santa Cruze Inc. (CA, USA).

### Assessment of IHC staining

To score the immunostaining of target proteins as we published previously [2], the intensity of IHC was classified into four categories—0, 1, 2 and 3—corresponding to no staining, weak staining, moderate staining and strong staining, respectively. The percentage of positively staining tumor cells was classified into five categories—0, 1, 2, 3 and 4—which corresponding to < 10, 10–25, 26–50, 51–75 and > 75%, respectively. The product of the staining intensity score and percentage of positive cells was considered the final score of target protein expression, which ranged from 0 (no staining) to 12 (75–100% of cells with 3 staining intensity scores).

### Evaluation of HIF-1α

HIF-1α was scored according to the presence of nuclear staining as previously published [2]. Only cells with completely and darkly stained nuclei were interpreted as positive expression. Additionally, because of the narrow range of the staining intensity, HIF-1α was only scored as 1+ and 0 according to the presence and absence of nuclear expression, respectively.

### Animal experiments

Five- to 6-week-old, 18–22 g male BALB/c mice were purchased from the Experimental Animal Center of Tongji Medical College, Huazhong University of Science and Technology. All studies involving mice were approved by Animal Care and Use Committee of Huazhong University of Science and Technology. Animals were housed in microisolator cages under pathogen-free conditions, in a controlled environment of temperature at 25 °C and 12-h cycles of light and dark. Mice were fed a standard laboratory diet and water ad libitum for at least 1 week to ensure proper health before study initiation. According to our published method [5], before implantation, H22 cells were injected and grown i.p. for 7 days in mice. After the mice were killed by cervical dislocation, H22 cells in ascites were collected. Cell viability was determined by trypan blue exclusion. Only preparations with > 90% viability of the cells were used. Each BALB/c was inoculated with 0.2 mL H22 tumor cell suspension (1 × 10^6^/mL PBS) by s.c. injection to the right flank. At the time of autopsy on day 14 after inoculation, tumors were dissected. The general conditions of mice including activity, tumor growth and weight were observed daily. The size of tumors was determined by caliper, measuring length (*L*) and width (*W*) of tumors every other day. Tumor volumes (V) were calculated by the formula: *V* = *L* × *W* ^2^ × 0.5.

### Cell culture and hypoxia exposure

The human HCC cell line HepG2 was purchased from ATCC and was cultured in DMEM media (Hyclone, USA), supplemented with 10% fetal bovine serum (Sijiqing, Zhejiang, China) at 37 °C in a humidified atmosphere containing 5% CO_2_, 21% O_2_. Hypoxia experiments were performed with a Hypoxia chamber (Coy Laboratory Products, Grass Lake, Michigan) at 1% O_2_, 37 °C in a 5% CO_2_ humidified environment. The cells were dissociated using 0.25% trypsin and 0.02% EDTA solution and resuspended into fresh medium once every 2–3 days.

### Measurement of cell proliferation with MTT assay

Cell proliferation was measured by 3-(4,5-dimethylthiazol-2-yl)-2,5-diphenyl tetrazolium (MTT, Sigma-Aldrich). Cells were seeded in a 96-well microplate (4500 cells/well) and incubated at 37 °C overnight before rHuEPO (R&D Systems, Minneapolis, USA) or soluble-EPOR (R&D Systems, Minneapolis, USA) treatments. After culture finished, the cells were incubated with a medium containing 5 mg/mL MTT for 4 h at 37 °C. After precipitated formazan was dissolved in 150 ul DMSO, then the absorbance was detected on a BioTek Elx 800 ELISA reader (Winooski, VT, USA) at a wavelength of 570 nm.

### Measurement of cell proliferation with flow cytometry

HepG2 cells were labeled with Carboxyfluorescein diacetate, succinimidyl ester (CFDA-SE/CFSE, final concentration: 1 μM, R&D Systems, Minneapolis, USA) for 7 min at 37 °C. Then, cells were washed and re-suspended in culture medium for additional 15 min to stabilize the CFSE staining. After the final wash step, cells were cultured in a 48-well microplate (5 × 10^4^ cells/well) overnight prior to rHuEPO (R&D Systems, Minneapolis, USA) or soluble-EPOR (R&D Systems, Minneapolis, USA) treatments. After being treated for the indicated times, cells were analyzed by flow cytometry.

### Western blot analysis

The cells were lysed in an RIPA buffer and centrifuged at 13,000 rpm for 15 min. Supernatants were collected, and the total protein concentration was quantified using BCA Protein Assay Kit (Beyotime Biotechnology, shanghai, China). Equal amounts of proteins were then separated by SDS-PAGE gels and transferred to a PVDF membrane. After blocking with 5% skim milk at room temperature for 1 h, the membranes were incubated with primary antibodies against EPO, EPOR or proliferating cell nuclear antigen (PCNA) (1:500). Equal lane loading was confirmed using a monoclonal antibody against β-actin (1:2000) (All of the above antibodies were procured from Santa cruz biotechnology, USA). The membranes were then incubated in an HRP-conjugated anti-rabbit IgG (1:2000) for 1 h at room temperature. Chemiluminescence was detected using an ECL Western blotting substrate, and band intensity was assessed using a gel imaging analysis system (Syngene, UK). The relative expression of target protein was normalized to the expression of β-actin.

### RNA purification and RT-PCR

Total RNA was isolated using Trizol reagent (Takara, Tokyo, Japan) following the manufacturer’s protocol. Reverse transcription of total RNA was performed using PrimeScript™II1st strand cDNA Synthesis Kit (Takara,Tokyo, Japan). PCR analysis was performed using the ABI Veriti96 (Applied Biosystems, Foster City, USA). The primers were shown in Table 1. The cycling parameters for PCR were as follows: denaturation at 95 °C 4 min, 94 °C 50 s, annealing at 54 °C, 56 °C, and 58 °C (for EPOR, GAPHD and EPO, respectively) for 30 s, and extension at 72 °C for 50 s for a total 30 cycles, which were followed by an extension at 72 °C for 10 min.

**Table 1:** Sequences of primers

Target gene	Conventional RT-PCR	Real-time PCR
*GAPDH*		
Forward	5′-CTCTGATTTGGTCGTATTGGG-3′	5′-ATTGCCCTCAACGACCACTT-3′
Reverse	5′-CTGGAAGATGGTGATGGGAT-3′	5′-CCCTGTTGGTCAACTCTTCC-3′
*EPO*		
Forward	5′-ATCACGACGGGCTGTGCTGAACAC-3′	5′-CCCTGTTGGTCAACTCTTCC-3′
Reverse	5′-ATCACGACGGGCTGTGCTGAACAC-3′	5′-GTGTACAGCTTCAGCTTTCC-3′
*EPOR*		
Forward	5′- GGGAGATGGCTTCCTTCTGGGCTC-3′	5′-GCACCGAGTGTGTGCTGAGCAA-3′
Reverse	5′-CGGGGACAGATGATGAGG-3′	5′-GGTCAGCAGCACCAGGATGAC-3′

### Real-time PCR

Target gene and internal control gene were measured on a 7300 Real-time PCR system using SYBR Green Master mix (TOYOBO, Osaka, Japan) according to the manufacturer’s protocol. GAPDH was served as the internal control. The primers were shown in Table 1. All the reactions were run in triplicate. The ^ΔΔ^Ct method was used for relative quantification of target gene expression levels. The results are expressed as fold change over control values.

### Immunofluorescence staining

Cells were fixed with 4% paraformaldehyde, permeabilized with 0.1% Triton X-100/PBS for 5 min.

After blocking with 2% FBS in PBS, cells were incubated with the primary antibodies against EPOR, for 1 h at room temperature. Secondary antibodies were goat anti–rabbit labeled with TRITC (Jackson ImmunoResearch Laboratories, Inc.) for 1 h. Nuclei were counterstained with 4,6-diamidino-2-phenylindole (Sigma, USA). Confocal images were acquired with a TCS S2 microscope adapted to a DMIRBE inverted microscope (Leica Microsystems, Inc.).

### EPOR binding assay

The EPOR binding studies were performed with ^125^I-EPO (Amersham Piscataway, NJ) as previously described [25]. After cultured under normoxia or hypoxia for indicated time, cells were then washed with binding buffer (PBS with calcium and magnesium with 1 mg/mL human serum albumin and 0.02% sodium azide). After cells were incubated with 5 nmol/L [^125^I] rHuEPO at room temperature for 3 h, binding was terminated by the addition of ice-cold wash buffer (PBS with calcium and magnesium with 1% fetal bovine serum). Nonspecific binding of [^125^I]-rHuEPO was determined by adding a 300-fold excess of unlabeled rHuEPO to the binding assay. Finally, cells were lysed with lysis buffer (20 mmol/L HEPES with 1% Triton, 10% glycerol and 0.1 mg/mL BSA) and surface-bound radioactivity was quantified using a γ-counter.

### Statistical methods

All of the data were analyzed by Graphpad Prime 5 software. All cell experiments were repeated at least three times. Differences between groups were evaluated using 2-tailed Student’s t test. The IHC score of the target protein was compared using non-parametric approach (Wilcoxon signed-rank test) between the tumor tissue and paired ANLTs in human or tumor tissue and paired liver tissue. *p* < 0.05 was considered to indicate a statistically significant difference. Spearman rank correlation was applied to assess the correlation among the protein level of EPO, EPOR, HIF-1α, and Ki-67.

## Results

### Expression of EPO and EPOR is up-regulated in human HCC tissue

**Fig. 1:**
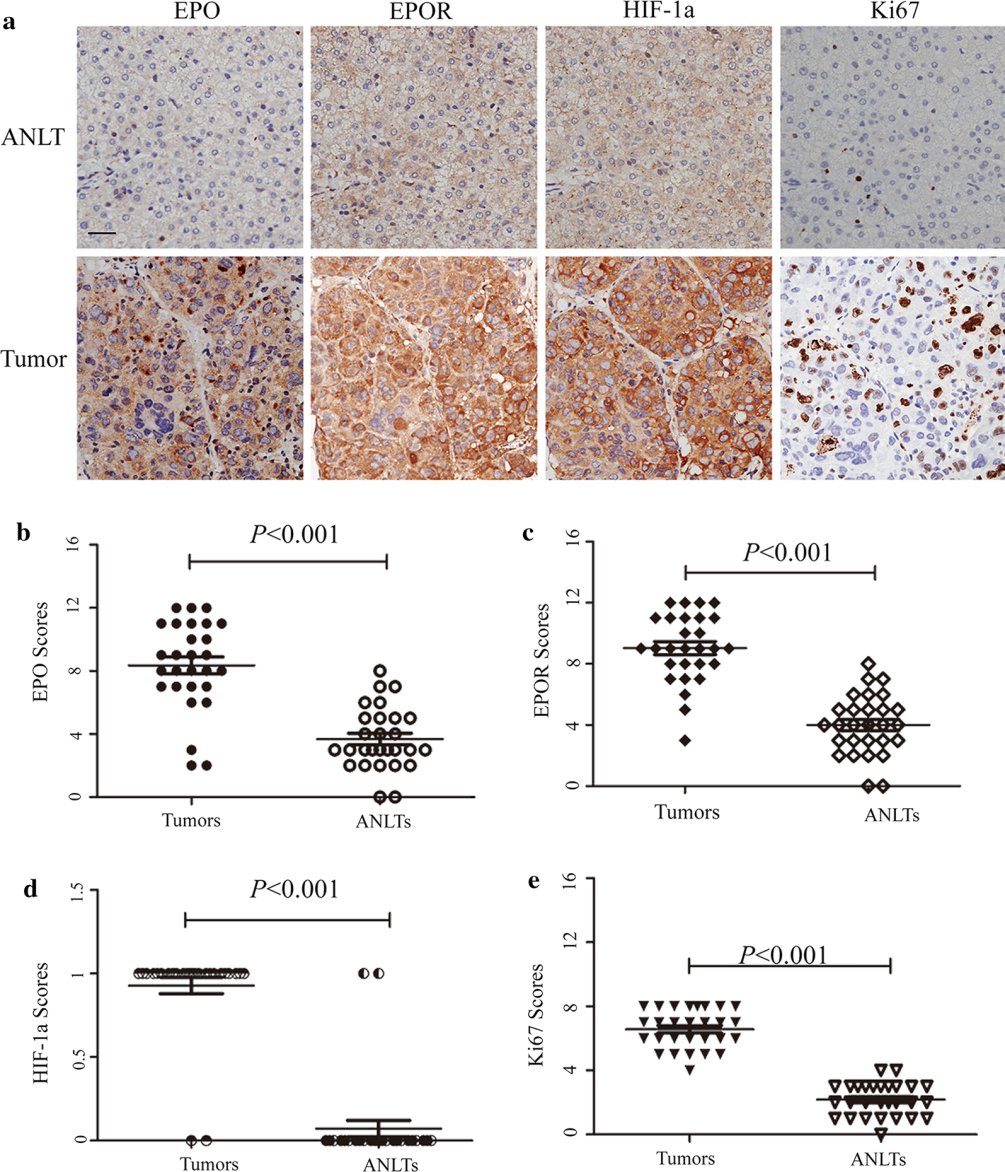
Expression of EPO, EPOR, HIF-1α and Ki-67 in human HCC tissues. **a** Representative images were taken. Scale bar, 200 μm. **b**–**e** IHC scores were displayed by scatter plot with the mean value indicated

Twenty-eight HCC patients accepted surgery and the tissues were sent to EPO and EPOR detection by IHC. Meanwhile, ANLTs from the same patient were set as the control group. It was found that EPO was nearly undetectable in ANLTs, but EPOR showed very weak staining (seen in Fig. 1a). While, in HCC tissue, both EPO and EPOR were strongly expressed. The mean score of EPO in tumor tissues was much higher than that in ANLTs, 8.357 ± 0.528 vs 3.679 ± 0.371 (*p* < 0.001, Wilcoxon signed rank test). Similarly, the mean scores of EPOR in tumor tissues and ANLTs were 9.036 ± 0.423 and 4.000 ± 0.371, respectively (*p* < 0.001, Wilcoxon signed rank test). As for the localization, different with EPO, which is predominantly in the cytoplasm, EPOR could be seen both in the cytoplasm and membrane.

### Correlations among EPO, EPOR, HIF-1α and Ki-67 in human HCC tissue

As the best known hypoxia-regulated gene, EPO is induced mainly by the activation of HIF-1α pathway [26–28]. In order to investigate whether there is a correlation between EPO/EPOR and HIF-1α in HCC, IHC was also applied to measure HIF-1α. Results showed a significant enhancement of its expression in HCC tissue compared with ANLTs. The mean score was elevated from 0.071 ± 0.050 to 0.929 ± 0.050 (*p* < 0.001, Wilcoxon signed rank test). The similar expression pattern was also observed in Ki-67, a commonly used tumor cell proliferation index. As shown in Fig. 1a, e, its mean score was increased from 2.179 ± 0.193 to 6.571 ± 0.227 (*p* < 0.001, Wilcoxon signed rank test).

As presented in Table 2, by using the Spearman’s rank correlation test, it was confirmed that the positive correlations among these four proteins (EPO vs. EPOR, EPO vs. Ki-67, EPO vs. HIF-1α, EPOR vs. Ki-67, EPOR vs. HIF-1α, and Ki-67 vs. HIF-1α) were close (r = 0.592, 0.574, 0.606, 0.533, 0.602 and 0.586, respectively). This analysis indicated that in the tumor region, which had a higher level of HIF-1α, EPO and EPOR also expressed higher and cells grew faster.

**Table 2:** Correlations among the expression levels of EPO, EPOR, Ki67 and HIF-1α in human HCC tissue

	EPO	EPOR	Ki67	HIF-1α
HIF-1α				
r	0.606	0.602	0.586	–
p	*0.001*	*<* *0.001*	*0.001*	–
Ki67				
r	0.574	0.533	–	–
p	*0.001*	*0.003*	–	–
EPOR				
r	0.592	–	–	–
p	*0.001*	–	–	–
EPO				
r	–	–	–	–
p	–	–	–	–

Spearman’s rank correlation test was applied

Italic values indicate significance of *p* value (*p* < 0.01)

*r* Spearman’s rank correlation coefficient

### Expression of EPO and EPOR is up-regulated in H22-bearing mice

**Fig. 2:**
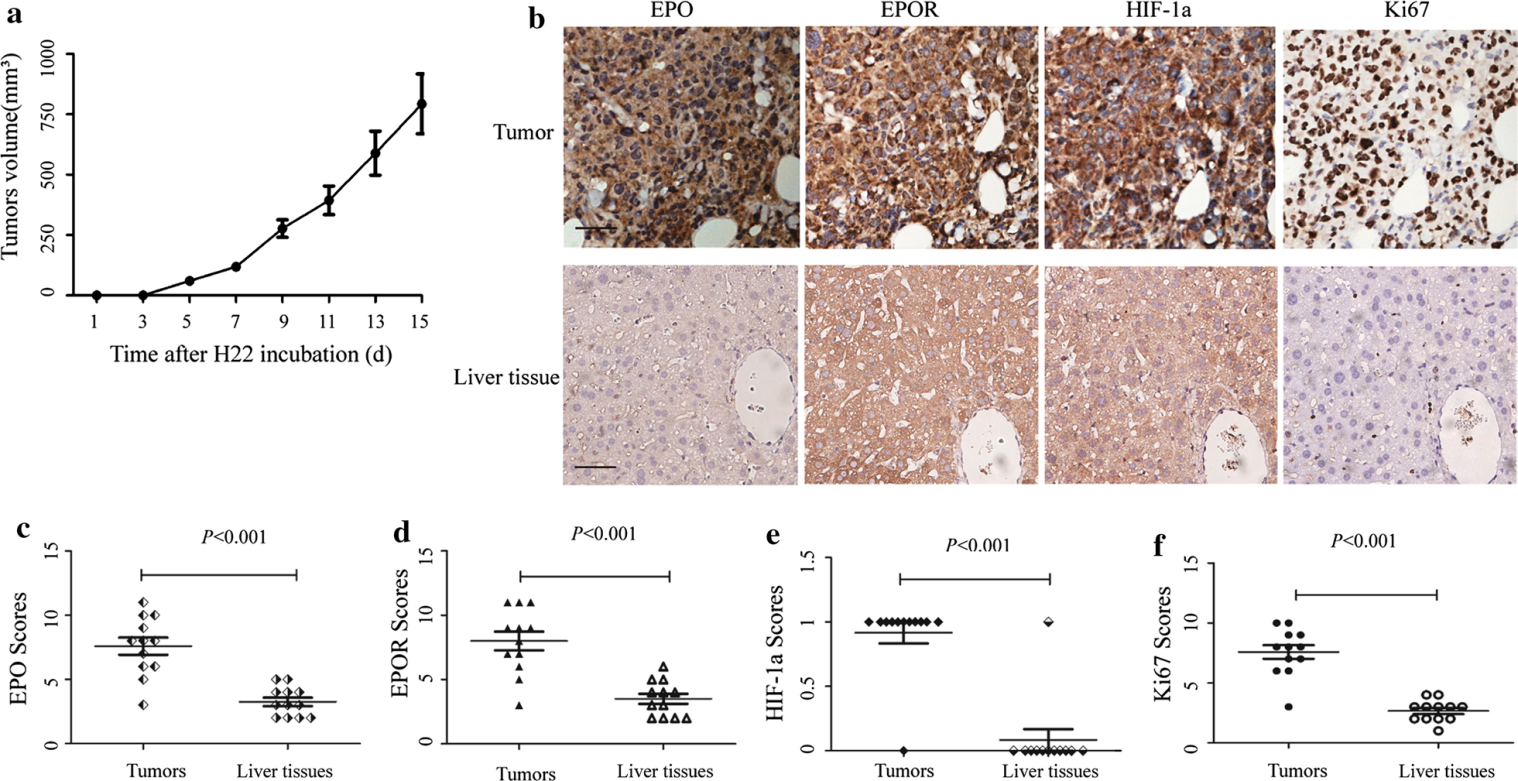
Expression of EPO, EPOR, HIF-1α and Ki-67 in HCC tumor from H22-bearing mice. 2 × 10^5^ H22 mouse tumor cells were inoculated s.c. into BALB/c mice. **a** The tumor growth was measured. The results were shown as the mean value from 12 mice. **b** Representative images were taken. Scale bar, 200 μm. **c**–**f** IHC scores were displayed by scatter plot with the mean value indicated

After confirming the up-regulation of EPO/EPOR in human HCC tissue and their positive correlation with HIF-1α and Ki-67, we applied a murine H22-bearing mice model to further evaluate the expression pattern and relationship among these proteins. After subcutaneous injection of H22 cells, the volume of tumor was measured and calculated once every 2 days. As seen in Fig. 2a, the tumors grew obviously, reaching an average volume of 793.1 ± 123.9 mm^3^ on day 14 post inoculation. This indicated a successful establishment of tumor-bearing mouse model.

At the time of autopsy (day 14 after inoculation), tumors were dissected and target proteins were measured with specific antibodies. Normal liver tissue from the same mouse was set as control. All of the four target proteins expressed much more obvious in tumor tissue than that in liver (Fig. 2b). The differences between the mean scores (in Table 3) were significant, with *p* value < 0.001 (shown in Fig. 2c–f and Table 3).

**Table 3:** IHC scores of four target proteins in HCC tumor tissue from H22-bearing mice (mean ± SEM)

Target protein	Score (n = 12)	
	Tumor tissue	Liver tissue
EPO	7.583 ± 0.668*	3.250 ± 0.329
EPOR	8.000 ± 0.728*	3.500 ± 0.399
HIF-1α	0.917 ± 0.083*	0.083 ± 0.083
Ki-67	7.583 ± 0.570*	2.667 ± 0.256

* *p* < 0.001 vs liver tissue

Positive correlations were also observed between these four proteins, except EPO and EPOR (shown in Table 4).

**Table 4:** Correlations among the expression levels of EPO, EPOR, Ki67 and HIF-1α in H22-bearing mice

	EPO	EPOR	Ki67	HIF-1α
HIF-1α				
r	0.624	0.624	0.731	–
p	*0.003*	*0.003*	*0.007*	–
Ki67				
r	0.873	0.766	–	–
p	*<* *0.001*	*0.004*	–	–
EPOR				
r	0.467	–	–	–
p	0.106	–	–	–
EPO				
r	–	–	–	–
p	–	–	–	–

Spearman’s rank correlation test was applied

Italic values indicate significance of *p* value (*p* < 0.01)

*r* Spearman’s rank correlation coefficient

### Hypoxia up-regulated the expression of EPO and EPOR in HepG2 cells

**Fig. 3:**
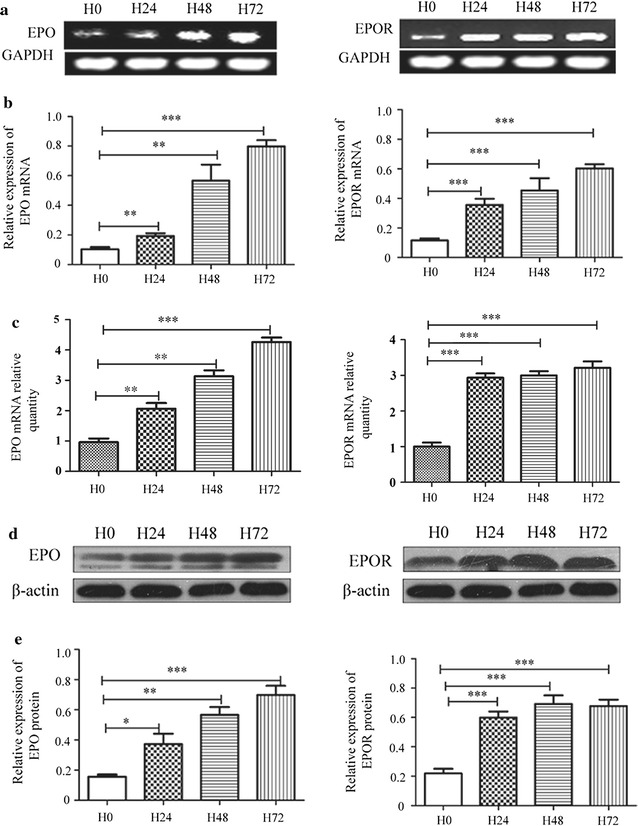
Expression of EPO and EPOR in HepG2 cells. **a** Expression of EPO and EPOR mRNA in HepG2 cells determined by regular RT-PCR and normalized to GAPDH mRNA in the same sample. **b** The histogram displays the ratio between the average level of target mRNA and internal control. **c** Expression of EPO and EPOR mRNA in HepG2 cells determined by real-time PCR. GAPDH was served as the housekeeping control gene. **d** Expression of EPO and EPOR protein in HepG2 cells. Total cell lysates were subjected to immunoblotting with specific antibody. β-actin serves as loading control. **e** the relative densities of EPO and EPOR. Results are representative of three independent experiments. H0, H24, H48 and H72 indicated cells cultured under hypoxia for 0, 24, 48 and 72 h, respectively. *p < 0.05 vs control, **p < 0.01 vs control and ***p < 0.001 vs control. Student’s t test is indicated

After confirmed the correlation between hypoxia and EPO/EPOR in clinical sample and mice model, we explored the effect of hypoxia on EPO and EPOR in HepG2 cells. Cells were cultured under 1% oxygen to imitate hypoxic micro-environment in tumor. 24–72 h hypoxia obviously enhanced nuclear HIF-1α protein level (data not shown) which indicates the successful establishment of cellular hypoxia. At the same time, hypoxia induced EPO and EPOR expression, both mRNA and protein in whole cell, with a time-dependent manner. As seen in Fig. 3a, b, after cultured in hypoxic condition for 72 h, the relative mRNA level of EPO and EPOR increased from 0.103 ± 0.009 to 0.798 ± 0.024 and 0.116 ± 0.008 to 0.602 ± 0.017, respectively (*p* < 0.001). Real-time PCR also presented a fourfold (4.260 ± 0.1514) and threefold (3.210 ± 0.1819) increase of EPO and EPOR at 72 h, respectively (Fig. 3c). Correspondingly, the relative protein level increased from 0.156 ± 0.014 to 0.700 ± 0.061 and 0.220 ± 0.031 to 0.677 ± 0.044, respectively (*p* < 0.001) (Fig. 3d, e).

### Hypoxia induced proliferation of HepG2 cells

Given the observed correlation between hypoxia and proliferation in vivo, we further investigated whether the in vitro experiments had similar pattern and how EPO/EPOR was involved.

**Fig. 4:**
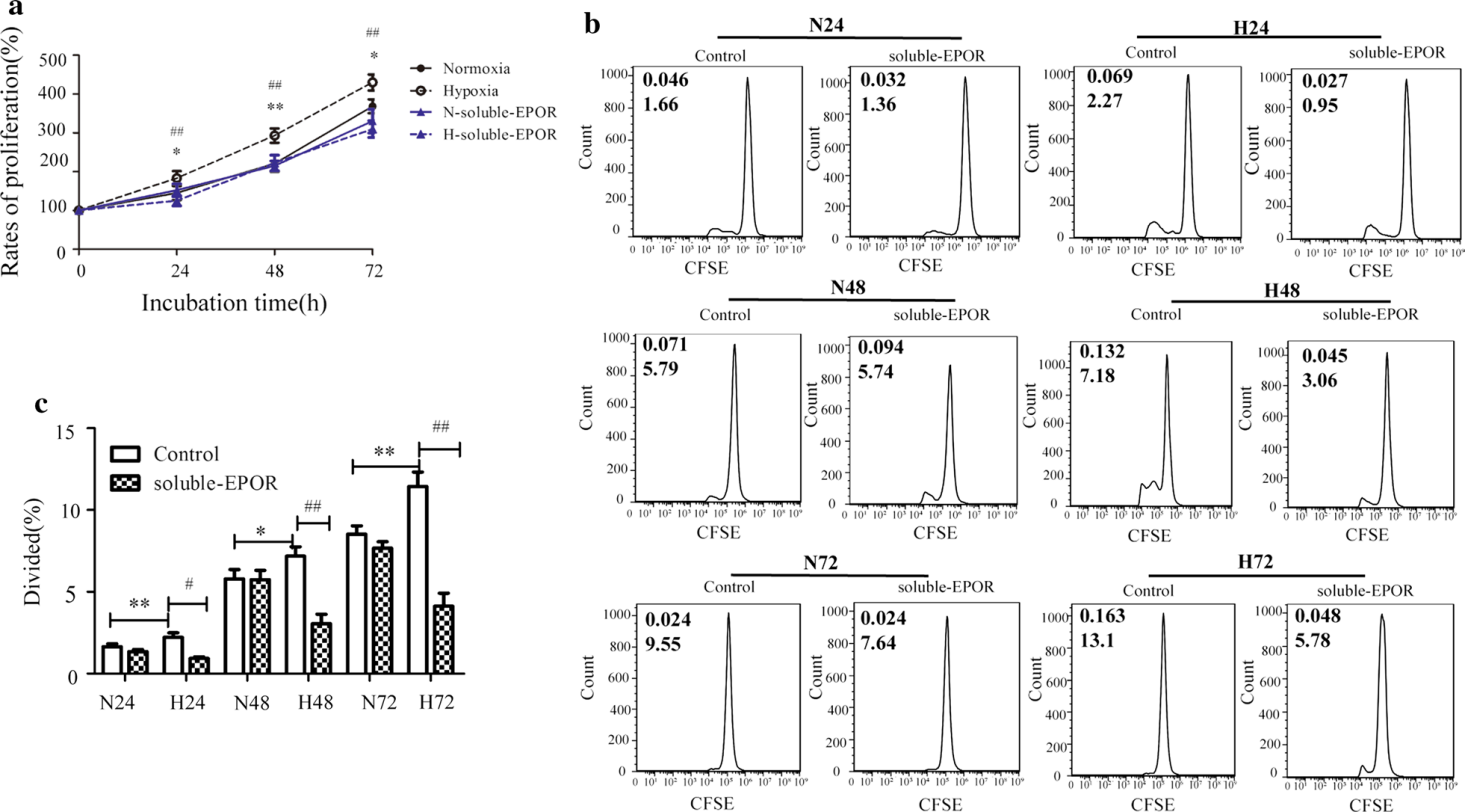
Hypoxia induced HepG2 cell proliferation through EPO/EPOR. **a** MTT assay. HepG2 cells were cultured under normal oxygen or hypoxia for 24, 48 and 72 h. *p < 0.05 hypoxia vs normoxia at the same time point, **p < 0.01 hypoxia vs normoxia at the same time point, ^##^ *p* < 0.01 hypoxia with soluble-EPOR vs hypoxia. **b** Histogram plots of CFSE fluorescence of cells cultured under normoxia or hypoxia with or without soluble-EPOR. The value (inset) for the percentage of cells that divided at least once (top left) and the average number of cell divisions (bottom left corner) are indicated for each sample. **c** Histograms of percentage of divided cells. N24, N48 and N72 indicated cells cultured for 24, 48 and 72 h, respectively. H24, H48 and H72 indicated cells cultured under hypoxia for 24, 48 and 72 h, respectively. Data shown are mean ± SEM of at least three independent experiments, each with three replicate wells. Student’s t test is indicated. *p < 0.05 hypoxia vs normoxia at the same time point, **p < 0.01 hypoxia vs normoxia at the same time point, ^#^ *p* < 0.05 hypoxia with soluble-EPOR vs hypoxia, ^##^ *p* < 0.01 hypoxia with soluble-EPOR vs hypoxia. Student’s t test is indicated

As observed in Fig. 4a, MTT assay indicated that cells grew obviously during the evaluation. But hypoxic cells had significantly higher proliferation rate at all time points. After continuously cultured for 72 h, HepG2 cells reached 368.57 ± 17.89 and 430.03 ± 20.52% of the initial counts, in normoxia and hypoxia group, respectively. Flow cytometry was further used to monitor the dilution of cellular label CFSE, which provided a measurement of both percentage of cells divided and the average number of cell divisions. The results also revealed that at the same time points, hypoxia cells had significantly higher percentage of divided cells (Fig. 4b, c).

### EPOR mediated hypoxia-induced HepG2 cell proliferation

When testing the role of EPO/EPOR pathway in hypoxia-induced HepG2 proliferation, cells were pre-treated with 0.5 μg/mL soluble-EPOR, which is known to antagonize the effect of EPO [29]. Both MTT assay and flow cytometry assay showed that, under normal oxygen, soluble-EPOR had no obvious effect on cell proliferation rate during 24–72 h. But, under hypoxia, it remarkably reduced the proliferation rate at all the time points. After cultured for 72 h, the percentage of cells divided was decreased from 12.670 ± 0.338 to 5.660 ± 0.753 (*p* < 0.01).

**Fig. 5:**
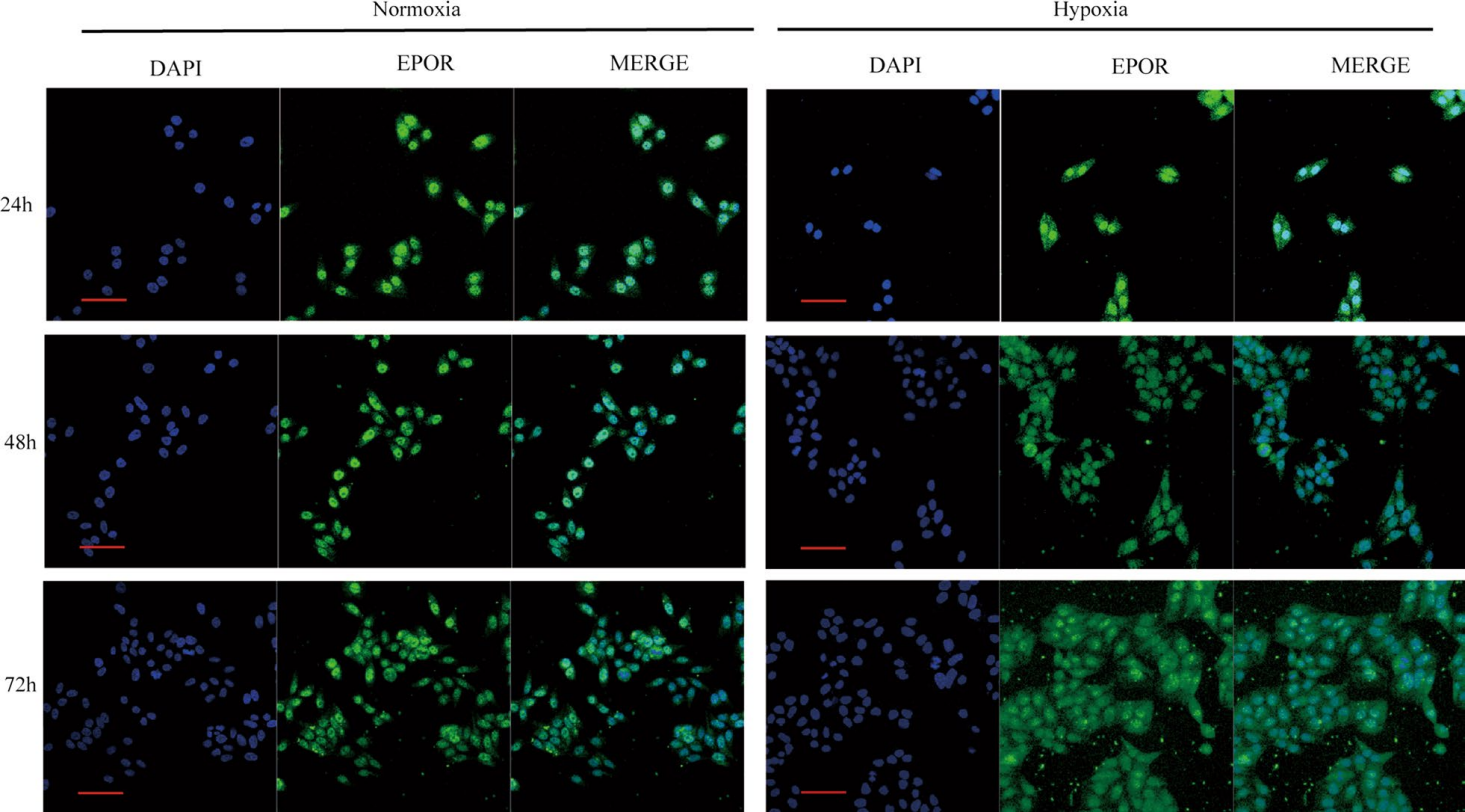
Expression and localization of EPOR in HepG2 cells. HepG2 cells were cultured under normoxia or hypoxia for 24, 48 and 72 h. The cells were analyzed by double immunofluorescence staining with anti-EPOR antibody (green) and DAPI for nuclei (blue). The results shown are representative of at least three different assessments. Scale bar: 50 μm

We also testified the cellular EPOR localization with Immunofluorescence staining. Seen in Fig. 5, in cells cultured under normoxia, the green fluorescence is well confined to the nucleus indicating a predominant location of EPOR in the nucleus. While, after treated with hypoxia, cells presented a uniform distribution of green fluorescence in nucleus, cytoplasm and membrane. The results indicated that EPOR obviously translocated from nucleus to cytoplasm and cell membrane under hypoxic condition. The results indicated that, hypoxia not only increased the total EPOR protein expression, but also induced its translocation and accumulation in the membrane.

Furthermore, to confirm the data obtained by Immunofluorescence staining and determine whether EPOR expression was functional at the cell surface, HepG2 cells were exposed to [^125^I]-rHuEPO with or without 300-fold excess of unlabeled rHuEPO. The results revealed that after exposure to hypoxia for 48 and 72 h, the EPO-specific binding activity was increased from 4230 ± 340 cpm to 12,000 ± 890 cpm and 15,340 ± 760 cpm, respectively (*p* < 0.05), which were obviously higher than the activity of cells under normoxia at the same time point.

### rHuEPO promoted the proliferation of HepG2 cells under hypoxia

**Fig. 6:**
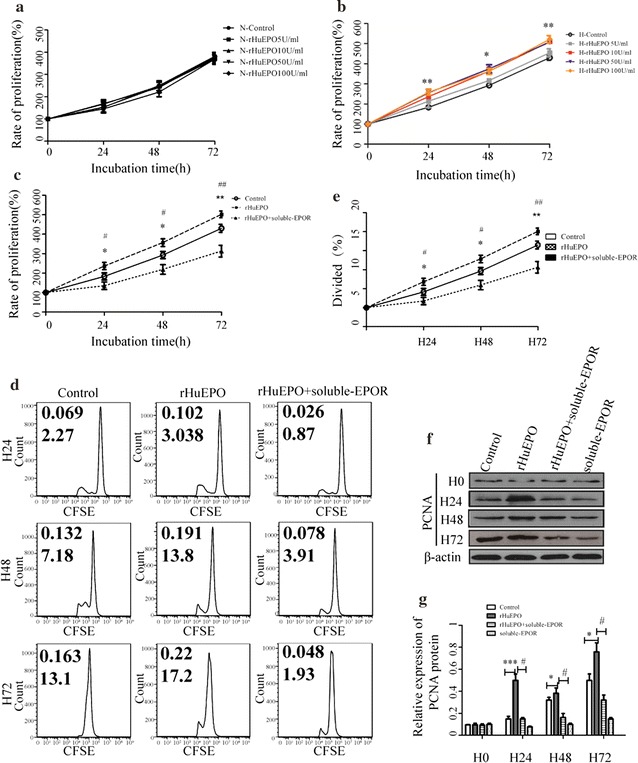
rHuEPO promoted HepG2 cells proliferation under hypoxia. **a**, **b** MTT assay. After 5, 10, 50 or 100 IU/mL rHuEPO was added into the cell culture media, HepG2 cells were cultured under normal oxygen (**a**) or hypoxia (**b**) for 24, 48 and 72 h. **p < 0.01 vs control at the same time point, ***p < 0.001 vs control at the same time point. **c**–**g** After HepG2 cells were treated with 10 IU/mL rHuEPO or/and 0.5 μg/mL soluble-EPOR, cells were cultured under hypoxia for 24, 48 and 72 h. **c** MTT assay. **d** Histogram plots of CFSE fluorescence of cells. The value (inset) for the percentage of cells that divided at least once (top left) and the average number of cell divisions (bottom left corner) are indicated for each sample. **e** Histograms of percentage of divided cells. Data shown are mean ± SEM of at least three independent experiments, each with three replicate wells. **f** Expression of PCNA protein in HepG2 cells. Total cell lysates were subjected to immunoblotting with specific antibody. β-actin serves as loading control. **g** The relative densities of PCNA. Results are representative of three independent experiments. H0, H24, H48 and H72 indicated cells cultured under hypoxia for 0, 24, 48 and 72 h, respectively. *p < 0.05 rHuEPO vs control, **p < 0.01 rHuEPO vs control at the same time point, ***p < 0.001 rHuEPO vs control, ^#^ *p* < 0.05 rHuEPO + soluble-EPOR vs rHuEPO at the same time point, ^##^ *p* < 0.01 rHuEPO + soluble-EPOR vs rHuEPO at the same time point. Student’s t test is indicated

After confirmed the correlation between endogenous EPO and HCC cell proliferation, we tested the effect of exogenous EPO. Different dose of rHuEPO was applied to stimulate the cells. At the concentration between 5 and 100 IU/mL, rHuEPO had no remarkable effect on cell proliferation under normoxia (Fig. 6a). But under 1% oxygen, 10, 50 and 100 IU/mL rHuEPO obviously elevated the cell proliferation rate (Fig. 6b). At 24, 48 and 72 h, the *p* values are lower than 0.001, 0.01 and 0.001 compared with control, respectively. In the following flow cytometry assay and PCNA detection, 10 IU/mL rHuEPO was chosen based on its most effective influence confirmed by MTT assay.

It was represented in Fig. 6d, e, that at all time points, the percentage of divided cells were significantly elevated by 10 IU/mL rHuEPO in hypoxic cells (*p* < 0.01). Similar results were also seen in Fig. 6f, g, which presents the PCNA protein level is upregulated by rHuEPO in HepG2 cells treated under 24, 48 or 72 h, reaching the peak value at 72 h.

The role of EPOR was further considered in the mechanism underlying which rHuEPO could promoted the hypoxic cell proliferation. Cells were pretreated with both rHuEPO and soluble-EPOR. Proliferation was tested with MTT, flow cytometry assay and PCNA protein. Results showed that cells treated with both rHuEPO and soluble-EPOR had lower proliferation rate, less percentage of cells divided and lower expression of PCNA (Fig. 6c–g).

## Discussion

ESAs, including rHuEPO, have been used since 1993 for the treatment of chemotherapy-induced anemia in Europe and the USA [30]. Although many clinical and preclinical researches have examined the benefits and risks associated with ESAs treatment, it is still inconclusive about whether this is a favorable therapy to the cancer patients with anemia, because the origin of cancer and the evaluation standard vary across studies. Several recent clinical trials have raised new concerns that ESAs may have direct tumor-stimulating effects and promote tumor progression, such as in head and neck cancer [22], breast cancer [31].

In patients with liver cancer following major hepatectomy, chemotherapy-dependent anemia might even lead to liver insufficiency, representing a serious challenge in liver surgery. Unfortunately, compared with other cancer types, very few studies have been conducted regarding the role of endogenous or exogenous EPO/EPOR in HCC. In this area, the initial investigation was approached with measurement of the serum EPO level. From 1993, there have been 3 clinical investigations reporting the elevated serum EPO value in a part of HCC patients [32–34]. It was speculated that this might be related to the abnormal production of EPO by liver tumor tissue, reduced hepatic clearance of EPO, or the influence of cytokine-mediated inflammatory factors. Direct intratumoral evidence was first presented in a case of a 64-year-old HCC patient in 2000, of whom tumor expressed higher amount of EPO [35]. This was supported by a larger scale clinical trial involving 50 patients [36]. Similarly, in our study, the up-regulation of EPO was also found in clinical samples. Meanwhile, with H22 tumor-bearing mice model, this phenomenon was further confirmed in vivo for the first time. It is very interesting that not only the HCC cells in situ but also the cells at metastatic place could express higher level of EPO, which was observed recently in a HCC patients with bone metastasis after liver segmentectomy [37].

Different with previous clinical study, hypoxia and proliferation index were further explored in our research. As we known, the rapidly expanding mass of tumor cell is inadequately oxygenated because of the diffusion limit of oxygen. HIF-1α is the key molecule to maintain the adaptive response of the cells to reduced oxygen level, making the cells capable of surviving under a hypoxic tumor microenvironment [38]. As the best known hypoxia-regulated gene, EPO is a critical factor through which HIF-1α mediates increased O_2_ delivery to hypoxic cells [26, 27]. In this study, with IHC assay, we found out that HIF-1α and Ki-67 protein were elevated in tumor tissue, both from HCC patients and mice model. In addition, Spearman rank correlation analysis revealed a strong positive relevance between EPO and these two proteins. That means in the tumor region, which had a higher HIF-1α level, EPO also expressed higher and cells grew faster. Although the overexpression of HIF-1α in HCC had been observed previously by us and several other groups [2, 5], this is the first time to reveal the correlation between EPO and hypoxia in HCC.

In addition to above correlation, in vitro experiments further revealed another relation between hypoxia and EPO. Hypoxia upregulated the expression of EPO in HepG2 cells at both transcript and protein levels. Although as early as 1998, Giovanardi et al. [39] hypothesized that an intratumoral hypoxia with compensatory production of EPO may have occurred in HCC patients, unfortunately, they did not present any evidence. First comparable in vitro evidence was reported on Hep3B cells. But the authors only measured the mRNA but not the protein of EPO [40]. In our study, it could be clearly seen that 24–72 h hypoxia obviously enhanced the expression of EPO accompanied with nuclear accumulation of HIF-1α. It could be easily acceptable that the effect of hypoxia is mediated by HIF-1α binding to the cis-acting DNA hypoxia response element and activating the transcription of EPO mRNA.

Although hypoxia is toxic to both normal cells and cancer cells, the later undergo genetic adaptive changes that allow them to survive and even proliferate under hypoxic conditions [41]. Based on the positive correlation we found between EPO, HIF-1α and Ki-67, a commonly used cell proliferation index, the effect of hypoxia and exogenous EPO on the cell proliferation was then tested. Through several different proliferation assays, the promotion of hypoxia on cell growth could be confirmed. The results also indicated that HepG2 cells treated with both hypoxia and rHuEPO had the highest proliferation rate among cells cultured under normoxia, normoxia with rHuEPO and hypoxia alone. What out of our expectation is that the proliferation promotion effect of exogenous rHuEPO could only be exerted under hypoxia but not normoxia. Previously, similar phenomena that HCC cells respond differently to EPO under different conditions was reported in the presence or absence of TGF-beta [42]. We are quite interested in the mechanism underlying which EPO could behave differently in different oxygen concentration.

EPOs, including rHuEPO, exerts their action through specific binding to EPOR which is a member of the cytokine receptor superfamily [43]. In recent years, it has become clear that the expression of EPOR was not strictly limited to erythroid or hematopoietic lineage. A variety type of non-hematopoietic cells, including endothelial cells, neurons, trophoblast cells and mammary epithelial cells could also expression EPOR [20]. But the data surrounding tumor cells, especially whether they have functional EPOR are still conflicting [44–48]. Some studies presented that even in the same cell type, there were different expression pattern between EPO mRNA and protein. It is believed that, besides the intrinsic diversity of cancer biology, the differences within EPOR protein detection results were mainly caused by the low-specificity of commercial antibody used, such as EPOR rabbit polyclonal, C-20, sc-695 (Santa Cruz) [49, 50]. In current study, we chose another antibody (M-20, sc-697, Santa Cruz) which was proved to have higher specificity and acceptance degree [51–53]. As a result, EPOR protein was clearly presented by IHC in tumor tissue from HCC patients and H22 tumor-bearing mice. More importantly, its expression was also positive correlated with HIF-1α and Ki-67.

**Fig. 7:**
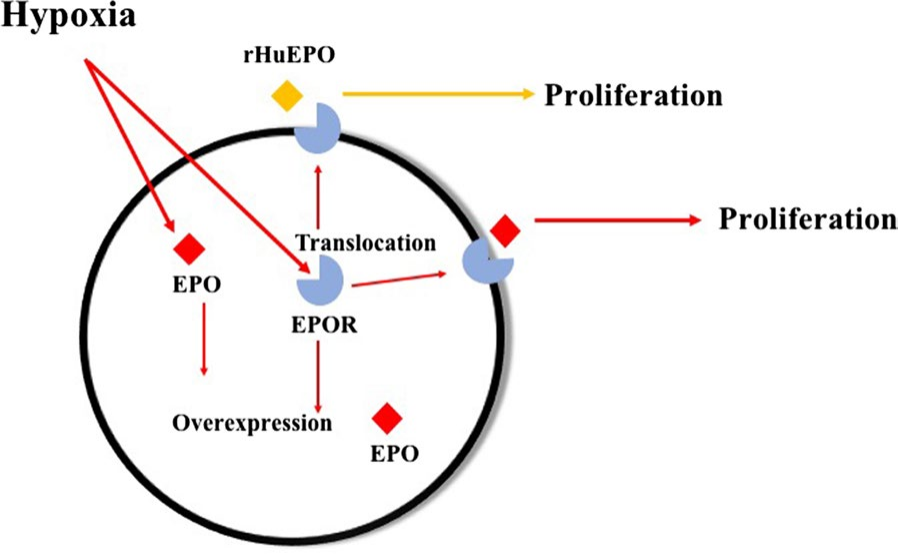
The possible mechanisms by which rHuEPO promoted HepG2 cells proliferation under hypoxia

As a well-reasoned decision, in order to make the role of EPOR in EPO-induced different proliferation status under normoxia or hypoxia, how hypoxia could influence the in vitro expression and location of EPOR was tested. HepG2 cells exhibited higher EPOR mRNA/protein expression and accumulation in the cell membrane after hypoxia stimulation. More importantly, EPO-specific binding activity was also higher in hypoxic cells, indicating a greater EPOR function. Combining the results from receptor blocking experiment with soluble EPOR, which not only inhibited hypoxia-induced proliferation but also inhibited the proliferation promotion effect of rHuEPO under hypoxia, we can get the conclusion that hypoxia-induced EPOR translocation mediated the effect of exogenous EPO on cell proliferation (Fig. 7). In details, hypoxia up-regulated the production of endogenous EPO and EPOR. It also induced the translocation of EPOR from nuclear and cytoplasm to membrane. Binding of EPOR to rHuEPO or endogenous generated EPO finally caused proliferation of cells. This can help us explain why rHuEPO had no obvious effect on cell proliferation under normoxia because cells were lack of functional EPOR. Actually, researchers had been attached the importance in the role of different EPOR location in tumor progression, such as in breast cancer cells [25]. A post hoc analysis suggested that progression-free survival was poorer in ESA-treated patients with EPOR-positive tumors [54].

It is well known, as one of the fundamental micro-environmental features of solid tumors, hypoxia plays a critical role in tumor-related cellular and physiologic events. Sustained hypoxia can enhance local and systemic malignant progression and aggressiveness. A lot of studies have been conducted to understand the underlying mechanism. Hypoxia-induced angiogenesis is one of the most important feature of solid tumors. This phenome is strongly correlated with EPO/EPOR and even progression of HCC patients [36, 43]. Our investigation provided new evidence that EPO/EPOR participated in the hypoxia-induced HCC proliferation. These results also will provide new possible targets for HCC diagnosis and treatment. It cannot be ignored that our data of protein expression of EPO and EPOR comes from tumor tissue and normal adjacent tissue. Although this is a commonly used setting of control, more and more researchers concern that tissue around tumor is suitable normal control. Because little is known about the transcriptomic profile of the normal adjacent tissue, how it is influenced by the tumor, and how the profile compares with non-tumor-bearing tissues. In this study, subcutaneous mice model was only established with H22 cell line. We believe that application of two different mice cell lines or establishment of xenograft model with human HCC cell line could provide us more information. There is still need for additional research to understand the full profile of this process.

## Conclusion

Our study demonstrated that EPO/EPOR were up-regulated in HCC and positively correlated with hypoxia and cell proliferation. At the same time, hypoxia-induced EPOR translocation mediated the effect of exogenous EPO on cell proliferation. These findings suggest that EPO/EPOR will be the possible targets for HCC diagnosis and treatment.

## Abbreviations

EPO: erythropoietin
HCC: hepatocellular carcinoma
ESA: erythropoiesis-stimulating agents
rHuEPO: recombinant human erythropoietin
ANLTs: adjacent non-tumor liver tissues
HIF-1α: hypoxia inducible factor-1α
MTT: 3-(4,5-dimethylthiazol-2-yl)-2,5-diphenyl tetrazolium
CFSE: carboxyfluorescein diacetate, succinimidyl ester
PCNA: proliferating cell nuclear antigen

## References

[1] Forner A, Llovet JM, Bruix J (2012). Hepatocellular carcinoma. Lancet.

[2] Ma M, Hua S, Li G, Wang S, Cheng X, He S, Wu P, Chen X (2017). Prolyl hydroxylase domain protein 3 and asparaginyl hydroxylase factor inhibiting HIF-1 levels are predictive of tumoral behavior and prognosis in hepatocellular carcinoma. Oncotarget.

[3] Ye SL, Chen X, Yang J, Bie P, Zhang S, Liu F, Liu L, Zhou J, Dou K, Hao C (2016). Safety and efficacy of sorafenib therapy in patients with hepatocellular carcinoma: final outcome from the Chinese patient subset of the GIDEON study. Oncotarget.

[4] Xiao H, Zhang B, Mei B, Zuo C, Wei G, Wang R, Zhang B, Chen X (2015). Hepatic resection for hepatocellular carcinoma in patients with portal hypertension: a long-term benefit compared with transarterial chemoembolization and thermal ablation. Medicine (Baltimore).

[5] Chen Y, Hao H, He S, Cai L, Li Y, Hu S, Ye D, Hoidal J, Wu P, Chen X (2010). Lipoxin A4 and its analogue suppress the tumor growth of transplanted H22 in mice: the role of antiangiogenesis. Mol Cancer Ther.

[6] Marmont AM (1997). Erythropoietin: biochemical characteristics, biologic effects, indications and results of use in hematology. Tumori.

[7] Abri Aghdam K, Soltan Sanjari M, Ghasemi Falavarjani K (2016). Erythropoietin in ophthalmology: a literature review. J Curr Ophthalmol.

[8] Szygula Z, Lubkowska A, Giemza C, Skrzek A, Bryczkowska I, Dolegowska B (2014). Hematological parameters, and hematopoietic growth factors: EPO and IL-3 in response to whole-body cryostimulation (WBC) in military academy students. PLoS ONE.

[9] Alural B, Duran GA, Tufekci KU, Allmer J, Onkal Z, Tunali D, Genc K, Genc S (2014). EPO mediates neurotrophic, neuroprotective, anti-oxidant, and anti-apoptotic effects via downregulation of miR-451 and miR-885-5p in SH-SY5Y neuron-like cells. Front Immunol.

[10] Kimakova P, Solar P, Solarova Z, Komel R, Debeljak N (2017). Erythropoietin and its angiogenic activity. Int J Mol Sci.

[11] Todosenko NM, Shmarov VA, Malashchenko VV, Meniailo ME, Melashchenko OB, Gazatova ND, Goncharov AG, Seledtsov VI (2016). Erythropoietin exerts direct immunomodulatory effects on the cytokine production by activated human T-lymphocytes. Int Immunopharmacol.

[12] Lu H, Wu X, Wang Z, Li L, Chen W, Yang M, Huo D, Zeng W, Zhu C (2016). Erythropoietin-activated mesenchymal stem cells promote healing ulcers by improving microenvironment. J Surg Res.

[13] Caro JJ, Salas M, Ward A, Goss G (2001). Anemia as an independent prognostic factor for survival in patients with cancer: a systemic, quantitative review. Cancer.

[14] Lang E, Bissinger R, Qadri SM, Lang F (2017). Suicidal death of erythrocytes in cancer and its chemotherapy: A potential target in the treatment of tumor-associated anemia. Int J Cancer.

[15] Aapro M, Jelkmann W, Constantinescu SN, Leyland-Jones B (2012). Effects of erythropoietin receptors and erythropoiesis-stimulating agents on disease progression in cancer. Br J Cancer.

[16] Littlewood TJ, Bajetta E, Nortier JW, Vercammen E, Rapoport B, Epoetin Beta Hematology Study G (2001). Effects of epoetin alfa on hematologic parameters and quality of life in cancer patients receiving nonplatinum chemotherapy: results of a randomized, double-blind, placebo-controlled trial. J Clin Oncol.

[17] Vansteenkiste J, Pirker R, Massuti B, Barata F, Font A, Fiegl M, Siena S, Gateley J, Tomita D, Colowick AB (2002). Double-blind, placebo-controlled, randomized phase III trial of darbepoetin alfa in lung cancer patients receiving chemotherapy. J Natl Cancer Inst.

[18] Osterborg A, Brandberg Y, Molostova V, Iosava G, Abdulkadyrov K, Hedenus M, Messinger D, Epoetin Beta Hematology Study G (2002). Randomized, double-blind, placebo-controlled trial of recombinant human erythropoietin, epoetin Beta, in hematologic malignancies. J Clin Oncol.

[19] Bennett CL, Silver SM, Djulbegovic B, Samaras AT, Blau CA, Gleason KJ, Barnato SE, Elverman KM, Courtney DM, McKoy JM (2008). Venous thromboembolism and mortality associated with recombinant erythropoietin and darbepoetin administration for the treatment of cancer-associated anemia. JAMA.

[20] Acs G, Zhang PJ, McGrath CM, Acs P, McBroom J, Mohyeldin A, Liu S, Lu H, Verma A (2003). Hypoxia-inducible erythropoietin signaling in squamous dysplasia and squamous cell carcinoma of the uterine cervix and its potential role in cervical carcinogenesis and tumor progression. Am J Pathol.

[21] Trost N, Stepisnik T, Berne S, Pucer A, Petan T, Komel R, Debeljak N (2013). Recombinant human erythropoietin alters gene expression and stimulates proliferation of MCF-7 breast cancer cells. Radiol Oncol.

[22] Henke M, Laszig R, Rube C, Schafer U, Haase KD, Schilcher B, Mose S, Beer KT, Burger U, Dougherty C (2003). Erythropoietin to treat head and neck cancer patients with anaemia undergoing radiotherapy: randomised, double-blind, placebo-controlled trial. Lancet.

[23] Bruix J, Sherman M, Llovet JM, Beaugrand M, Lencioni R, Burroughs AK, Christensen E, Pagliaro L, Colombo M, Rodes J (2001). Clinical management of hepatocellular carcinoma. Conclusions of the Barcelona-2000 EASL conference. European Association for the Study of the Liver. J Hepatol.

[24] Li WF, Ou Q, Dai H, Liu CA (2015). Lentiviral-mediated short hairpin RNA knockdown of MTDH inhibits cell growth and induces apoptosis by regulating the PTEN/AKT pathway in hepatocellular carcinoma. Int J Mol Sci.

[25] LaMontagne KR, Butler J, Marshall DJ, Tullai J, Gechtman Z, Hall C, Meshaw A, Farrell FX (2006). Recombinant epoetins do not stimulate tumor growth in erythropoietin receptor-positive breast carcinoma models. Mol Cancer Ther.

[26] Semenza GL (1998). Hypoxia-inducible factor 1: master regulator of O_2_ homeostasis. Curr Opin Genet Dev.

[27] Semenza GL (1999). Regulation of mammalian O_2_ homeostasis by hypoxia-inducible factor 1. Annu Rev Cell Dev Biol.

[28] Weidemann A, Johnson RS (2008). Biology of HIF-1alpha. Cell Death Differ.

[29] Welsch T, Zschabitz S, Becker V, Giese T, Bergmann F, Hinz U, Keleg S, Heller A, Sipos B, Klingmuller U (2011). Prognostic significance of erythropoietin in pancreatic adenocarcinoma. PLoS ONE.

[30] Fujisaka Y, Sugiyama T, Saito H, Nagase S, Kudoh S, Endo M, Sakai H, Ohashi Y, Saijo N (2011). Randomised, phase III trial of epoetin-beta to treat chemotherapy-induced anaemia according to the EU regulation. Br J Cancer.

[31] Leyland-Jones B, Semiglazov V, Pawlicki M, Pienkowski T, Tjulandin S, Manikhas G, Makhson A, Roth A, Dodwell D, Baselga J (2005). Maintaining normal hemoglobin levels with epoetin alfa in mainly nonanemic patients with metastatic breast cancer receiving first-line chemotherapy: a survival study. J Clin Oncol.

[32] Sawabe Y, Iida S, Tabata Y, Yonemitsu H (1993). Serum erythropoietin measurements by a one-step sandwich enzyme linked immunosorbent assay in patients with hepatocellular carcinoma and liver cirrhosis. Jpn J Clin Oncol.

[33] Pirisi M, Fabris C, Falleti E, Soardo G, Toniutto P, Gonano F, Bartoli E (1993). Evidence for a multifactorial control of serum erythropoietin concentration in liver disease. Clin Chim Acta.

[34] Malaguarnera M, Bentivegna P, Di Fazio I, Laurino A, Romano M, Trovato BA (1996). Erythropoietin in hepatocellular carcinoma. Bull Cancer.

[35] Matsuyama M, Yamazaki O, Horii K, Higaki I, Kawai S, Mikami S, Higashino M, Oka H, Nakai T, Inoue T (2000). Erythrocytosis caused by an erythropoietin-producing hepatocellular carcinoma. J Surg Oncol.

[36] Ribatti D, Marzullo A, Gentile A, Longo V, Nico B, Vacca A, Dammacco F (2007). Erythropoietin/erythropoietin-receptor system is involved in angiogenesis in human hepatocellular carcinoma. Histopathology.

[37] Koida S, Kimura T, Matsuda Y, Inoue M, Shimizu Y (2014). A case of bone metastases from hepatocellular carcinoma presenting with erythrocytosis after hepatectomy. Gan To Kagaku Ryoho.

[38] Ruan K, Song G, Ouyang G (2009). Role of hypoxia in the hallmarks of human cancer. J Cell Biochem.

[39] Giovanardi P, Sacchetti C, Cameroni P, Grandi M (1998). Erythrocytosis in patients with hepatocarcinoma in alcoholic cirrhosis: ectopic production of erythropoietin?. Recenti Prog Med.

[40] Sugimachi K, Tanaka S, Taguchi K, Aishima S, Shimada M, Tsuneyoshi M (2003). Angiopoietin switching regulates angiogenesis and progression of human hepatocellular carcinoma. J Clin Pathol.

[41] Harris AL (2002). Hypoxia–a key regulatory factor in tumour growth. Nat Rev Cancer.

[42] Ehnert S, Freude T, Eicher C, Burkhardt B, Martinez Sanchez JJ, Neumann J, Muhl-Benninghaus R, Dooley S, Pscherer S, Nussler AK (2014). Darbepoetin inhibits proliferation of hepatic cancer cells in the presence of TGF-beta. Arch Toxicol.

[43] Yang Z, Sun B, Zhao X, Shao B, An J, Gu Q, Wang Y, Dong X, Zhang Y, Qiu Z (2015). Erythropoietin and erythropoietin receptor in hepatocellular carcinoma: correlation with vasculogenic mimicry and poor prognosis. Int J Clin Exp Pathol.

[44] Juul SE, Anderson DK, Li Y, Christensen RD (1998). Erythropoietin and erythropoietin receptor in the developing human central nervous system. Pediatr Res.

[45] Sinclair AM, Todd MD, Forsythe K, Knox SJ, Elliott S, Begley CG (2007). Expression and function of erythropoietin receptors in tumors: implications for the use of erythropoiesis-stimulating agents in cancer patients. Cancer.

[46] Jelkmann W, Bohlius J, Hallek M, Sytkowski AJ (2008). The erythropoietin receptor in normal and cancer tissues. Crit Rev Oncol Hematol.

[47] Laugsch M, Metzen E, Svensson T, Depping R, Jelkmann W (2008). Lack of functional erythropoietin receptors of cancer cell lines. Int J Cancer.

[48] Sinclair AM, Rogers N, Busse L, Archibeque I, Brown W, Kassner PD, Watson JE, Arnold GE, Nguyen KC, Powers S (2008). Erythropoietin receptor transcription is neither elevated nor predictive of surface expression in human tumour cells. Br J Cancer.

[49] Elliott S, Busse L, Bass MB, Lu H, Sarosi I, Sinclair AM, Spahr C, Um M, Van G, Begley CG (2006). Anti-Epo receptor antibodies do not predict Epo receptor expression. Blood.

[50] Brown WM, Maxwell P, Graham AN, Yakkundi A, Dunlop EA, Shi Z, Johnston PG, Lappin TR (2007). Erythropoietin receptor expression in non-small cell lung carcinoma: a question of antibody specificity. Stem Cells.

[51] Hu MC, Shi M, Cho HJ, Zhang J, Pavlenco A, Liu S, Sidhu S, Huang LJ, Moe OW (2013). The erythropoietin receptor is a downstream effector of Klotho-induced cytoprotection. Kidney Int.

[52] Ludwig LS, Gazda HT, Eng JC, Eichhorn SW, Thiru P, Ghazvinian R, George TI, Gotlib JR, Beggs AH, Sieff CA (2014). Altered translation of GATA1 in Diamond-Blackfan anemia. Nat Med.

[53] Sinclair AM, Coxon A, McCaffery I, Kaufman S, Paweletz K, Liu L, Busse L, Swift S, Elliott S, Begley CG (2010). Functional erythropoietin receptor is undetectable in endothelial, cardiac, neuronal, and renal cells. Blood.

[54] Henke M, Mattern D, Pepe M, Bezay C, Weissenberger C, Werner M, Pajonk F (2006). Do erythropoietin receptors on cancer cells explain unexpected clinical findings?. J Clin Oncol.

